# Supplementary material for: A recurrent kinase domain mutation in PRKCA defines chordoid glioma of the third ventricle
Source: Nat Commun. 2018 Feb 23;9:810. doi: 10.1038/s41467-018-02826-8 (PMC5824822; doi:10.1038/s41467-018-02826-8)
Supplement: Supplementary file 1 — Supplementary Information [file 41467_2018_2826_MOESM1_ESM.pdf]

**Supplementary Table 1.** Clinical characteristics of chordoid glioma patient cohort.

| Patient | Age at dx | Sex | Presenting symptoms | Treatment | Outcome |
|---|---|---|---|---|---|
| CG-UCSF-1 | 45 | F | headache and nausea | subtotal resection, external beam radiation | died of disease 7 months after dx |
| CG-UCSF-2 | 65 | F | cognitive decline | biopsy, external beam radiation | died of disease 14 months after dx |
| CG-UCLA-1 | 34 | M | hypogonadism | biopsy, external beam radiation | alive with stable disease at 53 months after dx |
| CG-UVA-1 | 48 | M | unknown | subtotal resection | unknown |
| CG-UVA-2 | 47 | M | unknown | subtotal resection | died of post-operative complications one week after dx |
| CG-JHH-1 | 34 | M | unknown | resection | unknown |
| CG-MSK-1 | 57 | F | none, incidentally found | gross total resection | alive with no evidence of disease at 13 years after dx |
| CG-MSK-2 | 37 | F | unknown | resection | unknown |
| CG-UCO-1 | 67 | M | hypothermia and dysequilibrium | biopsy followed by subtotal resection | alive with stable disease at 2 months after dx |
| CG-MGH-1 | 53 | F | unknown | resection | unknown |
| CG-NYU-1 | 45 | F | somnolence and cognitive decline | subtotal resection | unknown |
| CG-Egypt-1 | 49 | F | somnolence and headaches | biopsy | unknown |
| CG-Brazil-1 | 58 | M | unknown | resection | unknown |

**Supplementary Table 2.** Somatic *PRKCA* mutations identified in the 13 chordoid gliomas.

| Tumor | Chromosome | Position | Reference allele | Variant allele | Gene | Variant | Variant | Reference transcript | Tumor coverage | Tumor mutant allele frequency | Normal coverage | Normal mutant allele frequency |
|---|---|---|---|---|---|---|---|---|---|---|---|---|
| CG-UCSF-1 | chr17 | 64738741 | G | C | PRKCA | c.1387G>C | p.D463H | NM_002737 | 212 | 41% | 212 | 0% |
| CG-UCSF-2 | chr17 | 64738741 | G | C | PRKCA | c.1387G>C | p.D463H | NM_002737 | 458 | 19% | - | - |
| CG-UCLA-1 | chr17 | 64738741 | G | C | PRKCA | c.1387G>C | p.D463H | NM_002737 | 282 | 35% | 106 | 0% |
| CG-UVA-1 | chr17 | 64738741 | G | C | PRKCA | c.1387G>C | p.D463H | NM_002737 | 52 | 22% | - | - |
| CG-UVA-2 | chr17 | 64738741 | G | C | PRKCA | c.1387G>C | p.D463H | NM_002737 | 144 | 22% | - | - |
| CG-JHH-1 | chr17 | 64738741 | G | C | PRKCA | c.1387G>C | p.D463H | NM_002737 | 406 | 21% | - | - |
| CG-MSK-1 | chr17 | 64738741 | G | C | PRKCA | c.1387G>C | p.D463H | NM_002737 | 95 | 42% | - | - |
| CG-MSK-2 | chr17 | 64738741 | G | C | PRKCA | c.1387G>C | p.D463H | NM_002737 | 273 | 34% | - | - |
| CG-UCO-1 | chr17 | 64738741 | G | C | PRKCA | c.1387G>C | p.D463H | NM_002737 | 556 | 21% | - | - |
| CG-MGH-1 | chr17 | 64738741 | G | C | PRKCA | c.1387G>C | p.D463H | NM_002737 | 158 | 31% | - | - |
| CG-NYU-1 | chr17 | 64738741 | G | C | PRKCA | c.1387G>C | p.D463H | NM_002737 | 448 | 13% | - | - |
| CG-Egypt-1 | chr17 | 64738741 | G | C | PRKCA | c.1387G>C | p.D463H | NM_002737 | 59 | 12% | - | - |
| CG-Brazil-1 | chr17 | 64738741 | G | C | PRKCA | c.1387G>C | p.D463H | NM_002737 | 443 | 21% | - | - |

**Supplementary Table 3.** Chromosomal copy number alterations identified in the 13 chordoid gliomas.

| Tumor | Gains | Losses |
|---|---|---|
| CG-UCSF-1 | 1q, 8 | 6q |
| CG-UCSF-2 | none | none |
| CG-UCLA-1 | none | none |
| CG-UVA-1 | none | none |
| CG-UVA-2 | none | none |
| CG-JHH-1 | none | none |
| CG-MSK-1 | none | 18 |
| CG-MSK-2 | none | 1p |
| CG-UCO-1 | none | none |
| CG-MGH-1 | none | none |
| CG-NYU-1 | none | none |
| CG-Egypt-1 | none | none |
| CG-Brazil-1 | none | none |

CG-UCSF-1

CG-UCSF-2

CG-UCO-1

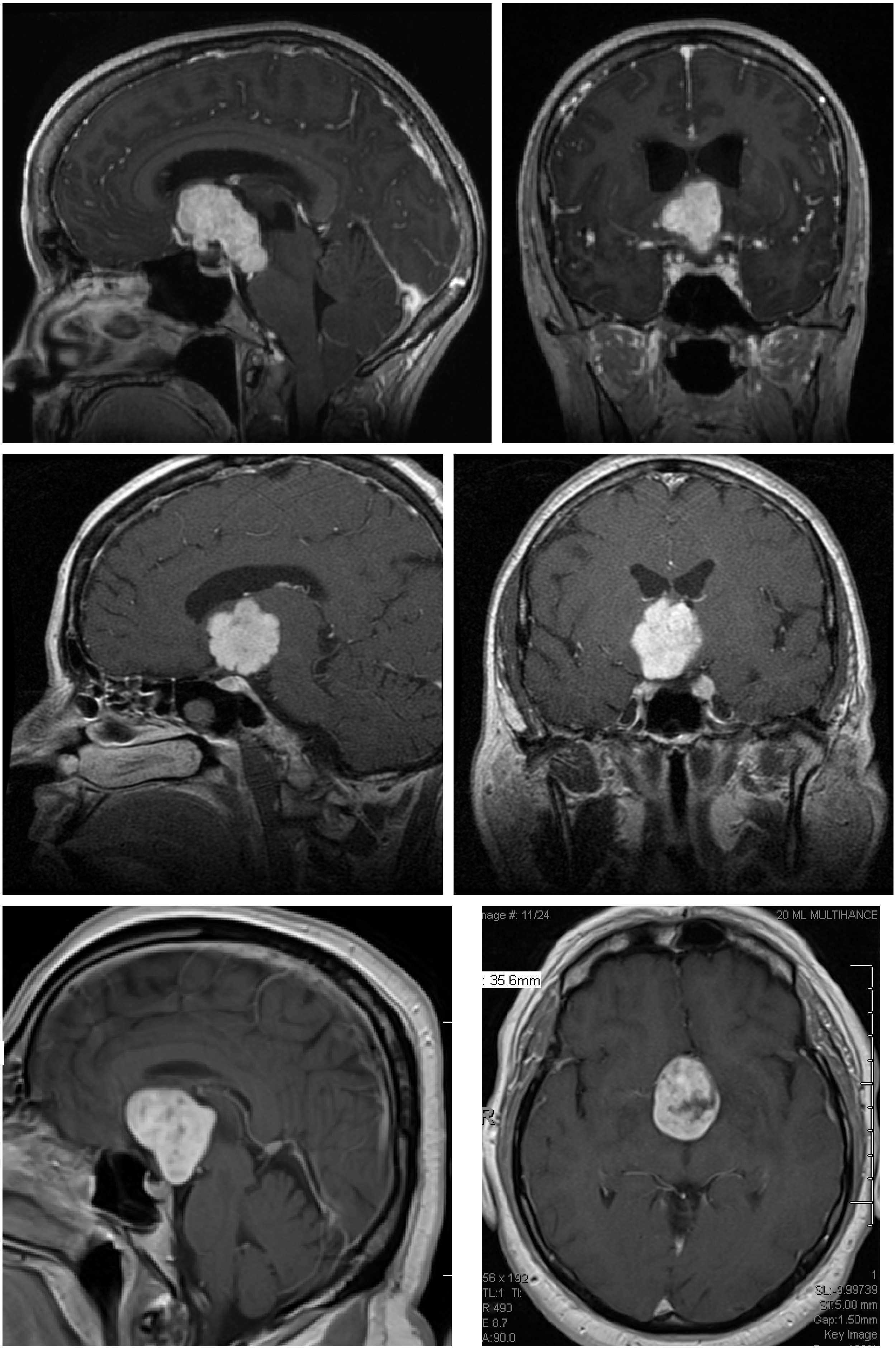


**Supplementary Figure 1.** Radiographic features of the chordoid gliomas. Shown are T1-weighted post-contrast magnetic resonance images demonstrating enhancing, well-circumscribed masses centered in the anterior third ventricle in all cases.

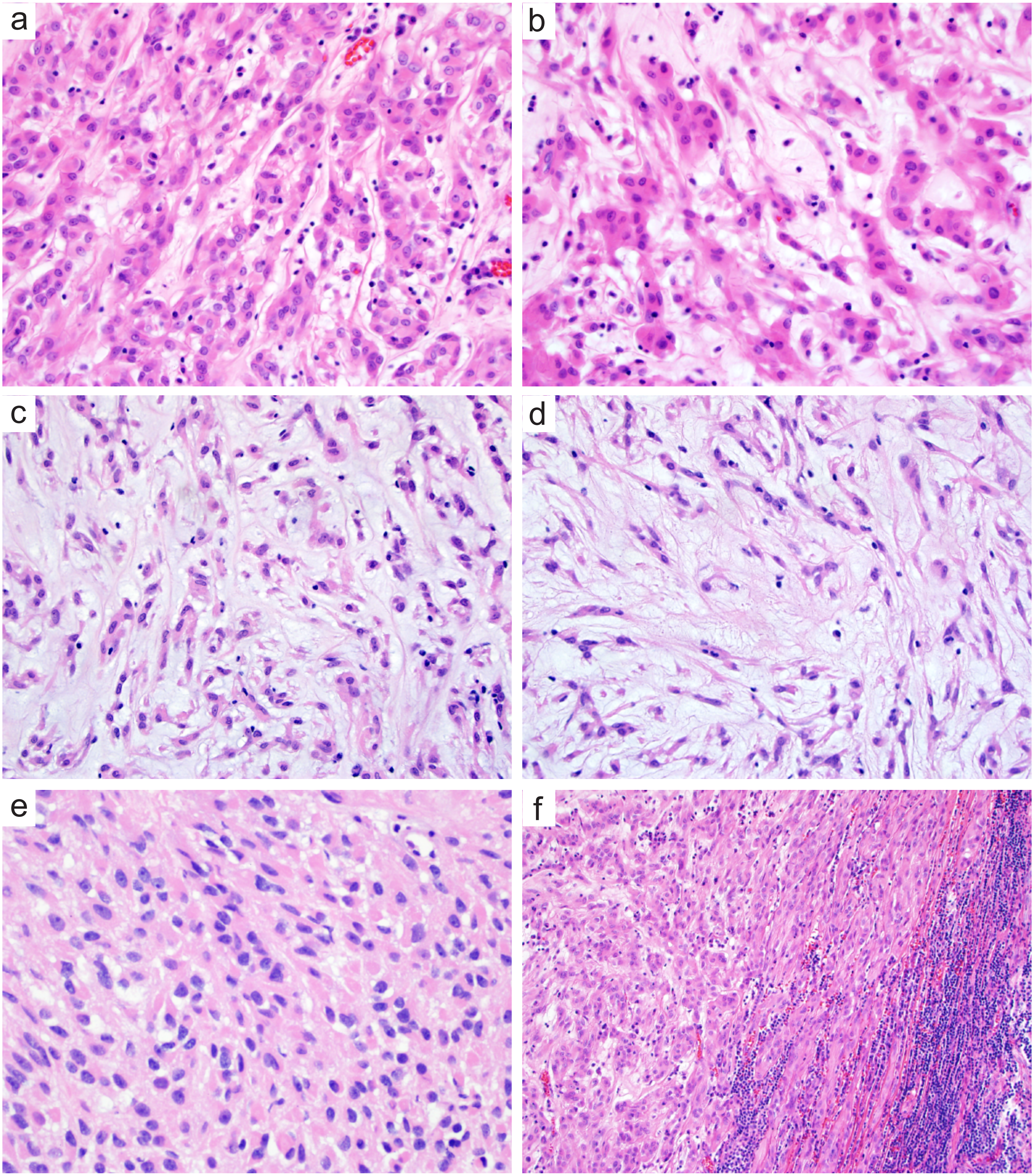


**Supplementary Figure 2.** Histologic features of the chordoid gliomas. (**a,b**) Tumors are characterized by cohesive cords and clusters of epithelioid cells with abundant eosinophilic cytoplasm in a mucin-rich stroma. Mitotic activity and nuclear atypia are minimal (CG-UVA-1). (**c,d**) Some tumors demonstrate more elongate to spindled morphology (CG-UCO-1). (**e**) Some tumors contain solid areas reminiscent of subependymal giant cell astrocytoma (CG-UCSF-1). (**f**) A common feature is the presence of a dense lymphoplasmacytic inflammatory infiltrate, often at the periphery of the tumor (CG-UVA-1).

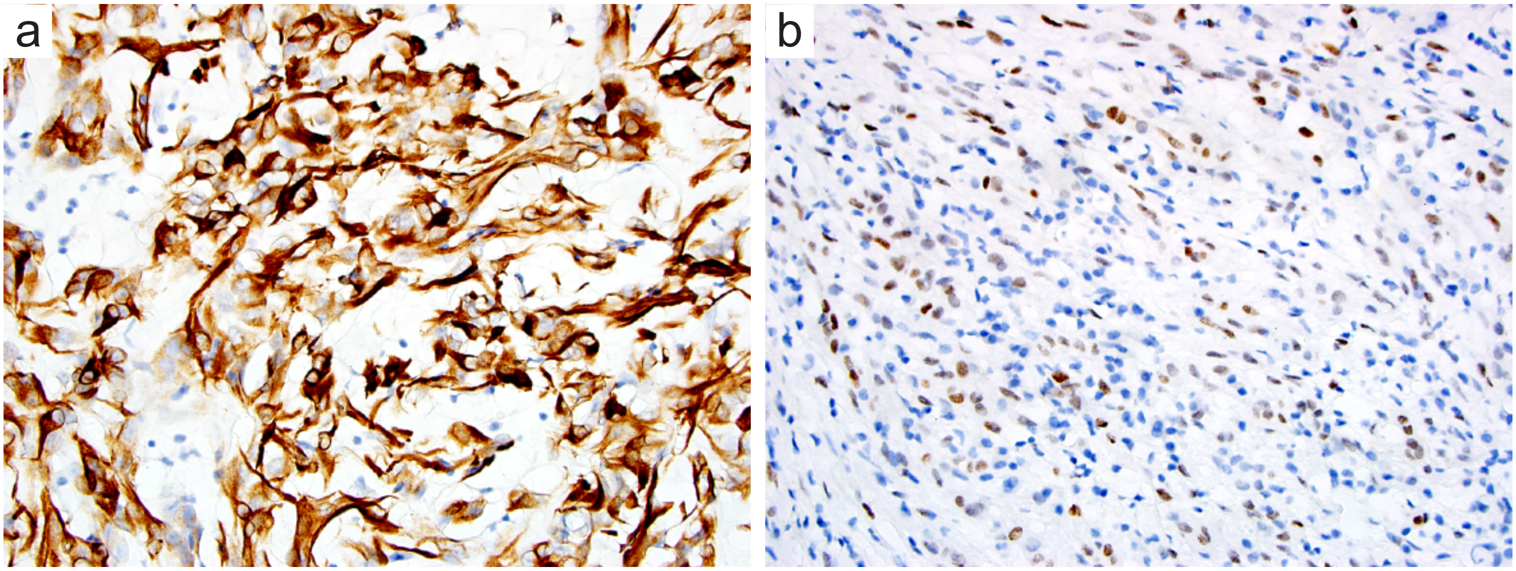


**Supplementary Figure 3.** Immunohistochemical features of the chordoid gliomas. (**a**) Diffuse positivity for glial fibrillary acidic protein (GFAP) in tumor cells. (**b**) Nuclear positivity for TTF-1 (also known as NKX2.1) in a subset of tumor cells.

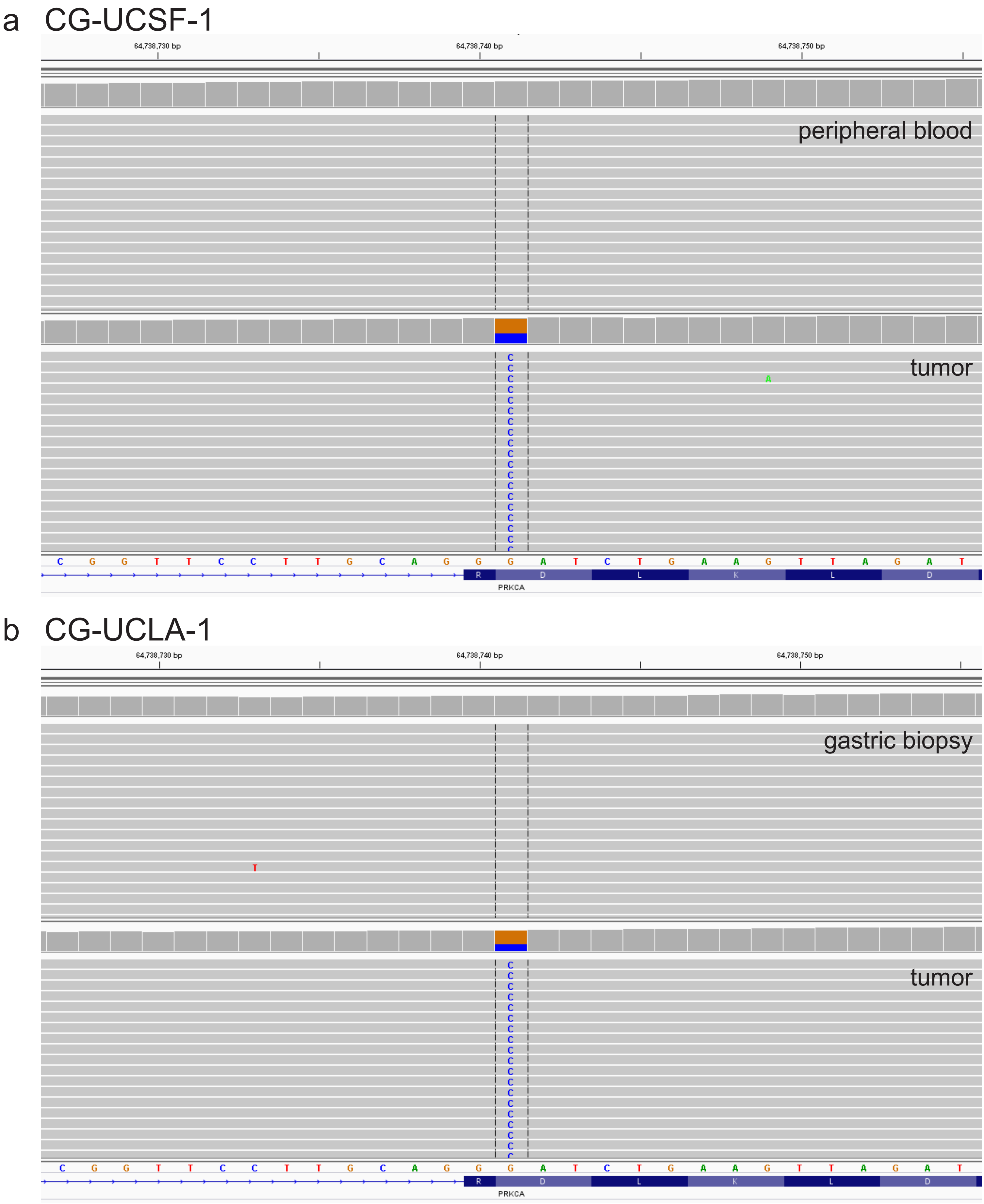


**Supplementary Figure 4.** A recurrent somatic *PRKCA* p.D463H mutation defines chordoid glioma of the third ventricle. Sequencing reads containing a G>C mutation in two tumor samples but not in matched normal tissue from the patients causing a p.D463H substitution. (**a**) Tumor CG-UCSF-1. (**b**) Tumor CG-UCLA-1.

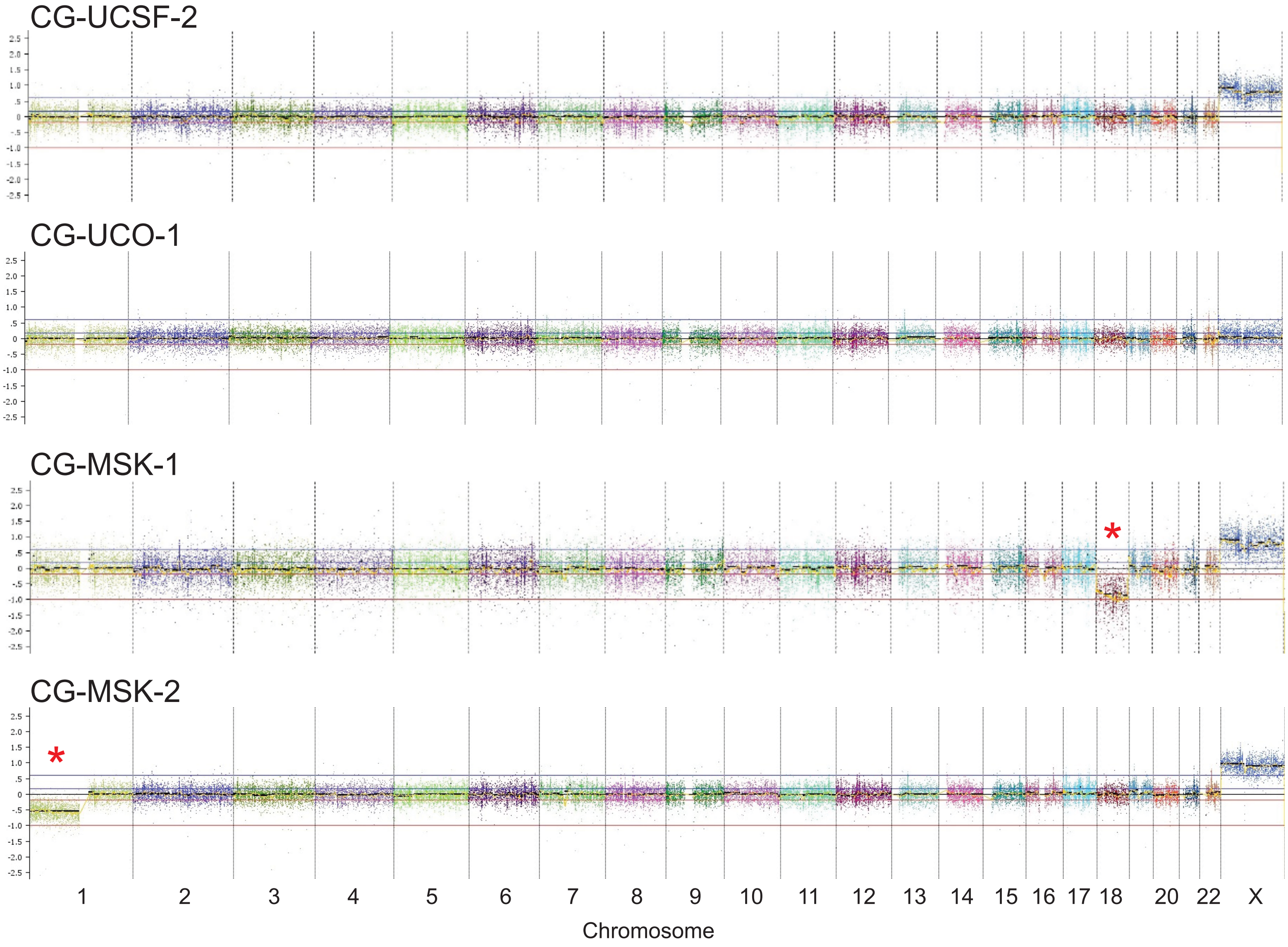


**Supplementary Figure 5.** Chromosomal copy number alterations in the 13 chordoid gliomas. Genome-wide copy number inferred from targeted next-generation sequencing data revealed a balanced diploid genome in 10 of the tumors including CG-UCSF-2 and CG-UCO-1. Tumor CG-MSK-1 demonstrated loss of chromosome 18. Tumor CG-MSK-2 demonstrated loss of chromosome 1p. Tumor CG-UCSF-1 demonstrated loss of chromosome 6q and gains of 1p and 8. No focal amplifications or deletions were identified in any of the tumors.

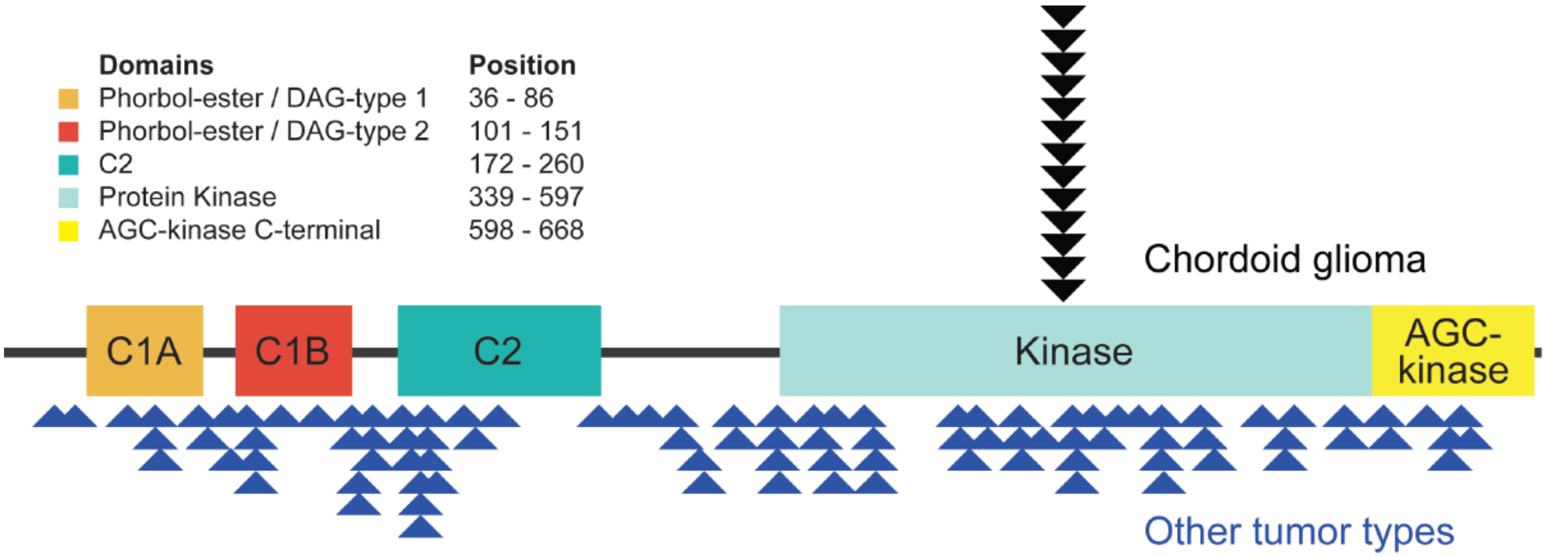


| Tumor Type | Non-synonymous somatic mutations |
|---|---|
| Adrenal gland | 0 / 613 (0.0%) |
| Autonomic ganglia | 0 / 766 (0.0%) |
| Biliary tract | 1 / 368 (0.3%) |
| Bone | 1 / 567 (0.2%) |
| Breast | 9 / 2327 (0.4%) |
| Cervix | 3 / 324 (0.9%) |
| Endometrium | 0 / 640 (0.0%) |
| Esophagus | 1 / 1105 (0.1%) |
| Hematopoietic | 1 / 3380 (0.0%) |
| Kidney | 2 / 1742 (0.1%) |
| Large intestine | 12 / 1594 (0.8%) |
| Liver | 3 / 1816 (0.2%) |
| Lung | 3 / 2283 (0.1%) |
| Oropharynx | 4 / 1238 (0.3%) |
| Ovary | 3 / 925 (0.3%) |
| Pancreas | 5 / 1766 (0.3%) |
| Pleura | 0 / 164 (0.0%) |
| Prostate | 4 / 1563 (0.3%) |
| Skin | 26 / 1215 (2.1%) |
| Soft tissue | 1 / 542 (0.2%) |
| Stomach | 7 / 824 (0.8%) |
| Thyroid | 0 / 697 (0.0%) |
| Urinary tract | 1 / 672 (0.1%) |

| CNS Tumor Type | Non-synonymous somatic mutations |
|---|---|
| Diffuse/anaplastic astrocytoma | 0 / 107 (0.0%) |
| Oligodendroglioma | 0 / 37 (0.0%) |
| Glioblastoma | 1 / 929 (0.1%) |
| Glioma NOS | 2 / 609 (0.3%) |
| Pilocytic astrocytoma | 0 / 10 (0.0%) |
| Pleomorphic xanthoastrocytoma | 0 / 12 (0.0%) |
| Ependymoma | 0 / 55 (0.0%) |
| Medulloblastoma | 1 / 499 (0.2%) |
| Atypical teratoid/rhabdoid tumor | 0 / 23 (0.0%) |

**Supplementary Figure 6.** Domain structure of the human PKC$\alpha$ protein with the location of the p.D463H mutation identified in each of the 13 chordoid gliomas (black arrowheads). Also shown are all confirmed somatic nonsynonymous mutations in the 30,367 human tumors with *PRKCA* sequencing data available in the version 81 release of the Catalogue of Somatic Mutations in Cancer (COSMIC) database (blue arrowheads).

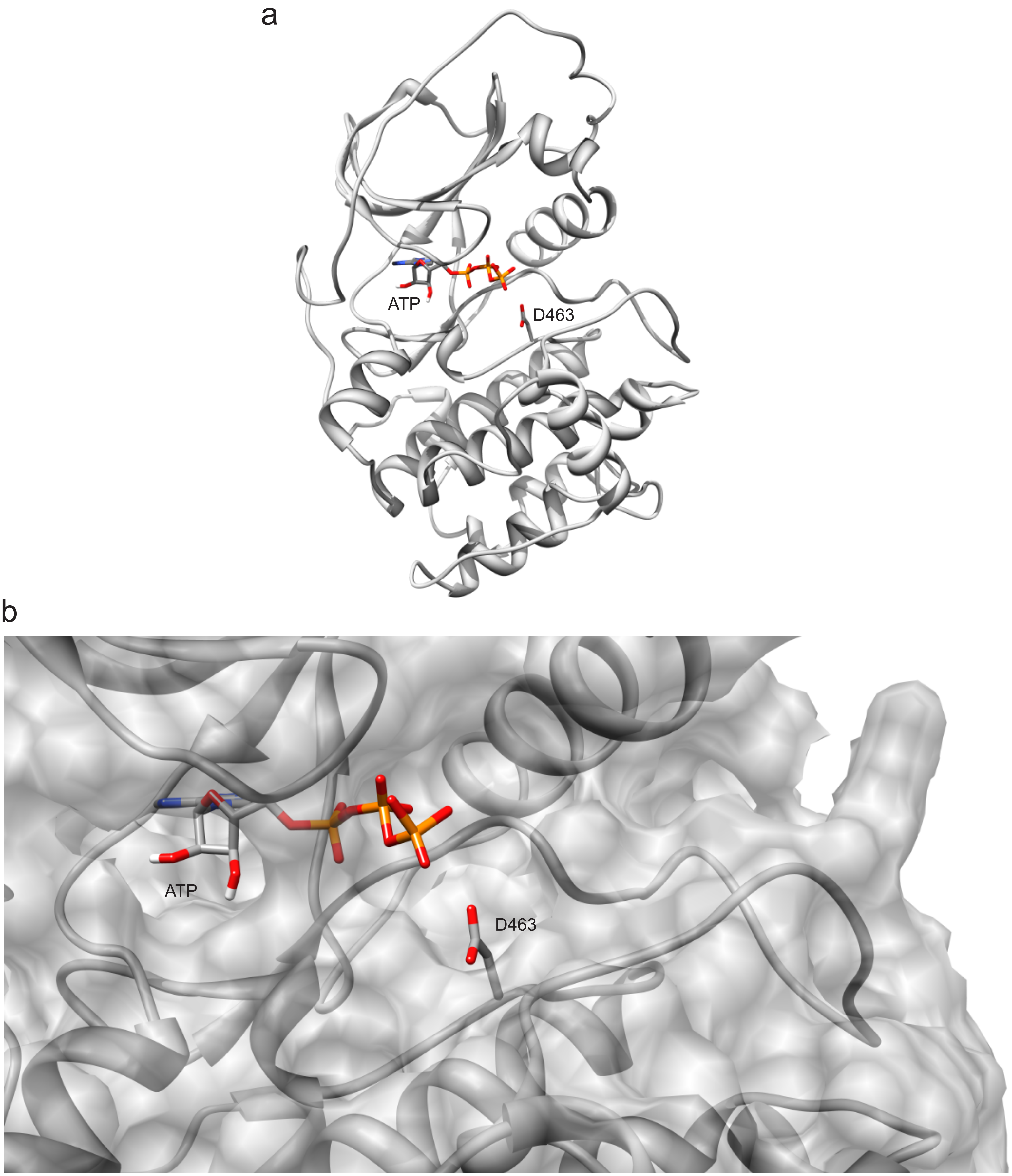


**Supplementary Figure 7.** Structural modeling of wildtype and D463H-mutant PKC$\alpha$.
(**a**) Model of PKC$\alpha$ with wildtype p.D463 (Asp463) and ATP bound within the active site of the kinase domian. (**b**) Solvent accesible surface area representation of PKC$\alpha$ focused on the active site of the kinase domain with wildtype p.D463 (Asp463) and bound ATP.

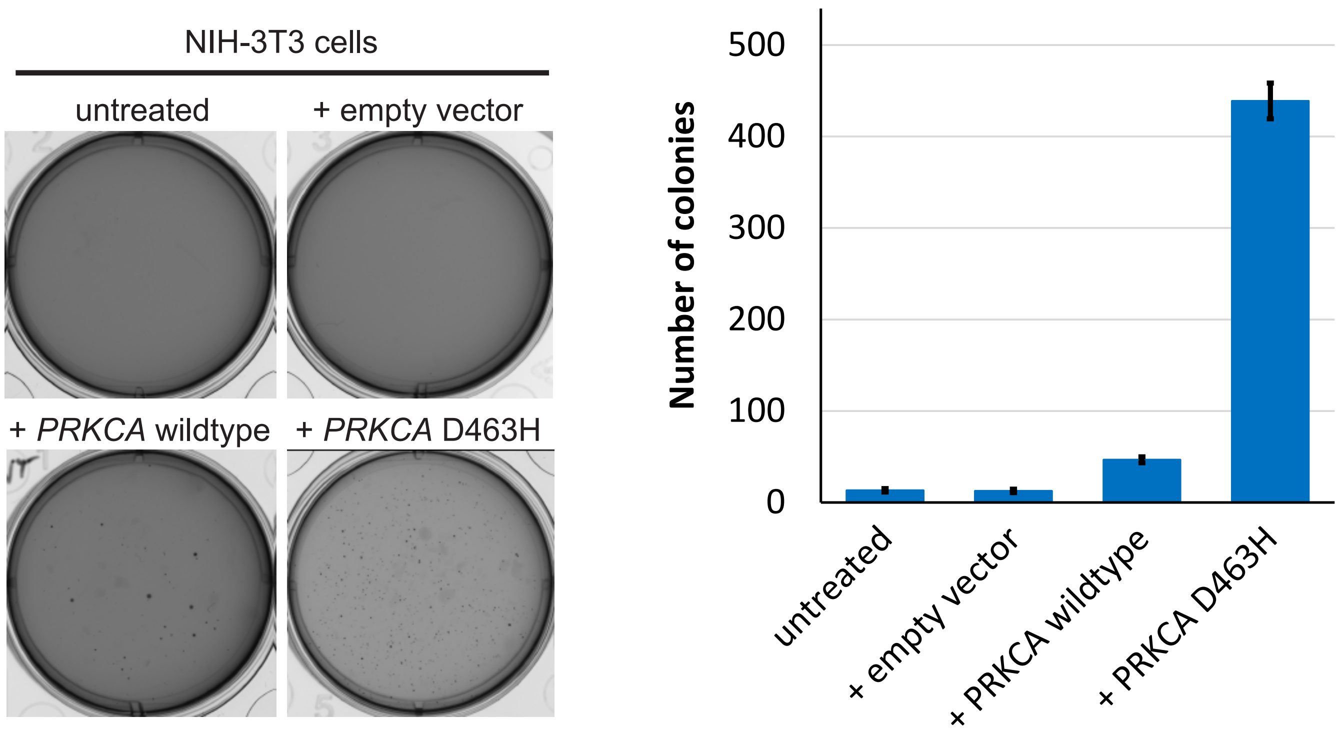


**Supplementary Figure 8.** Mutant *PRKCA* drives anchorage-independent growth of NIH-3T3 cells. NIH-3T3 cells were plated in soft agar 24 hours after lentiviral transduction with empty vector, wildtype *PRKCA*, or D463H-mutant *PRKCA*. Colonies were imaged and quantitated after 14 days. Error bars represent standard deviation from the mean of six replicates derived from two independent experiments performed in triplicate.

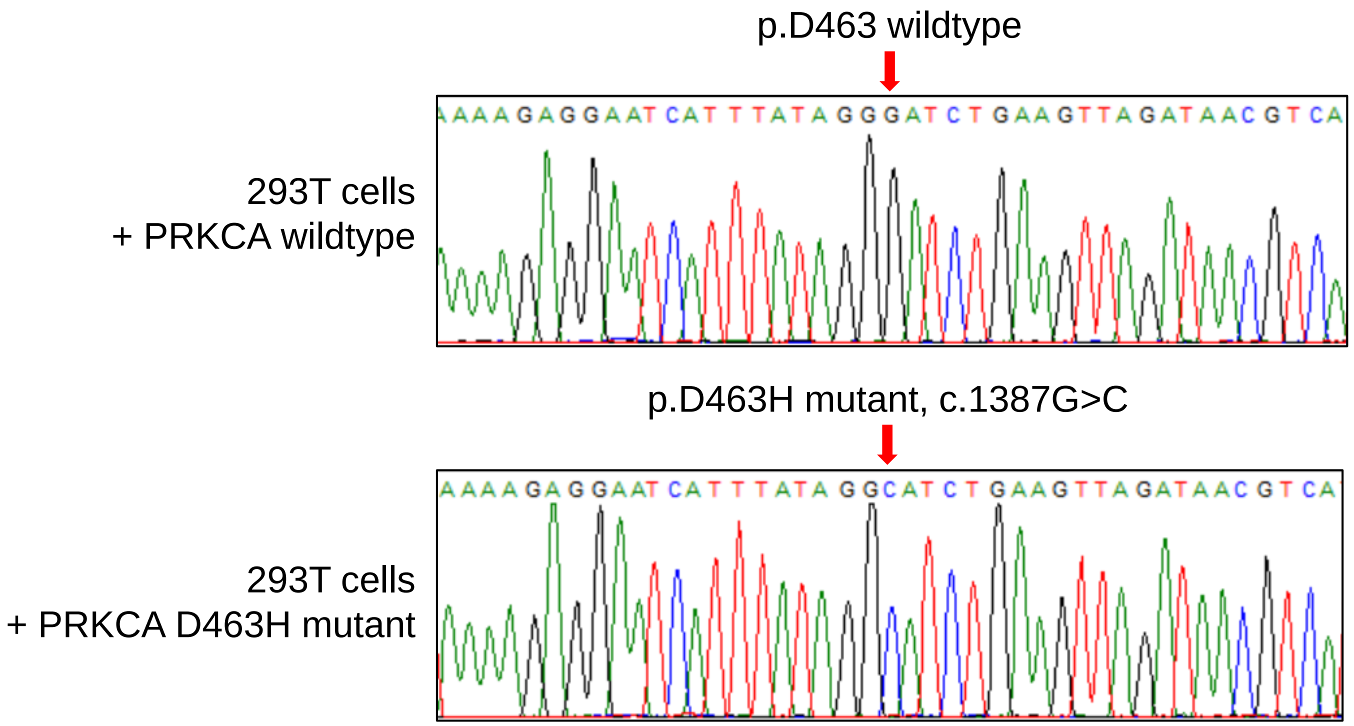


**Supplementary Figure 9.** Confirmation of wildtype and D463H mutant *PRKCA* mRNA expression after ectopic overexpression. Total RNA was isolated from 293T cells 48 hours after transient transfection with wildtype or D463H mutant *PRKCA* cDNA, and reverse transcription-PCR was performed to amplify *PRKCA* transcripts as described in the Methods. Shown are Sanger sequencing traces of these RT-PCR products.

**from Figure 2b**

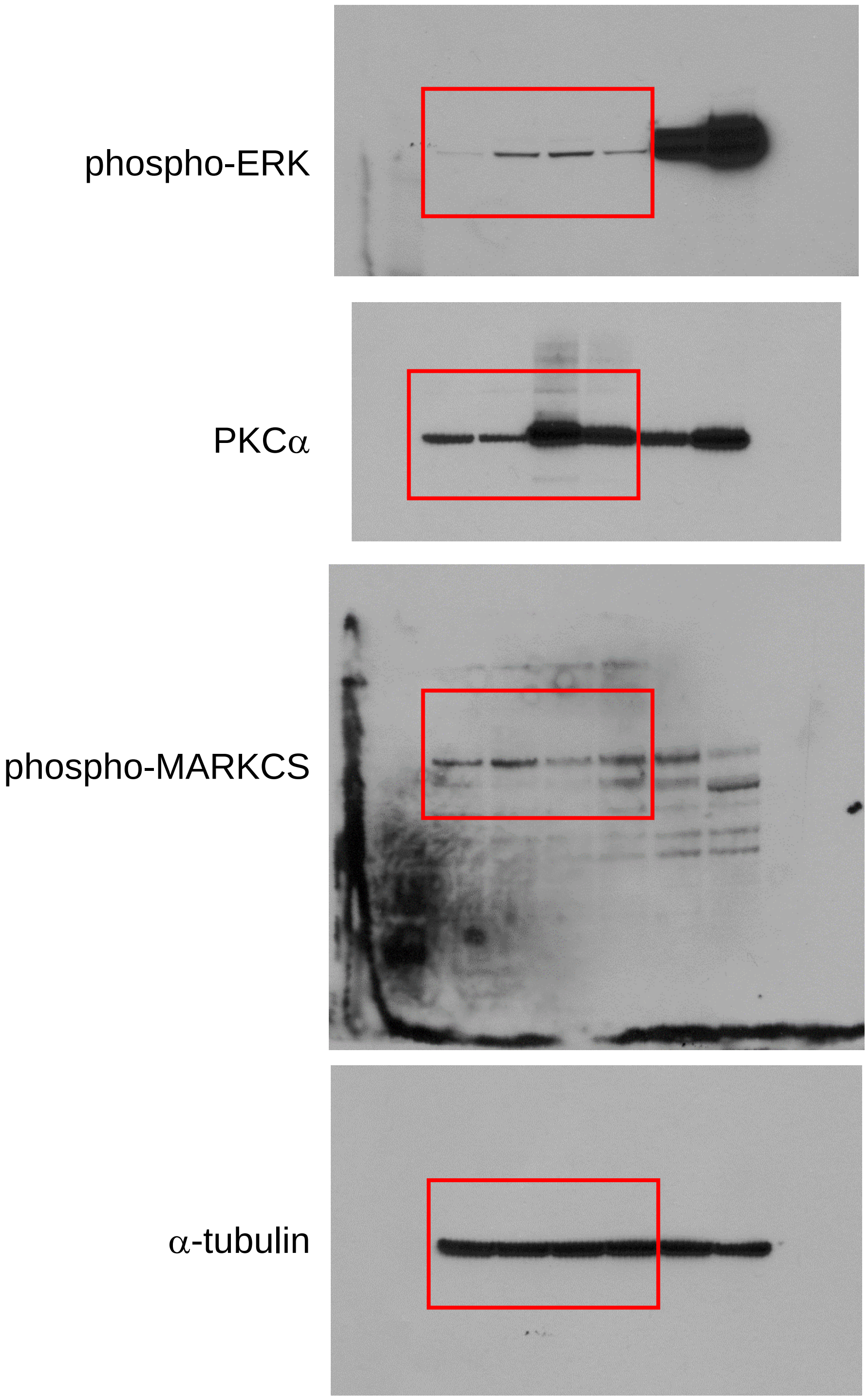

**from Figure 2c**

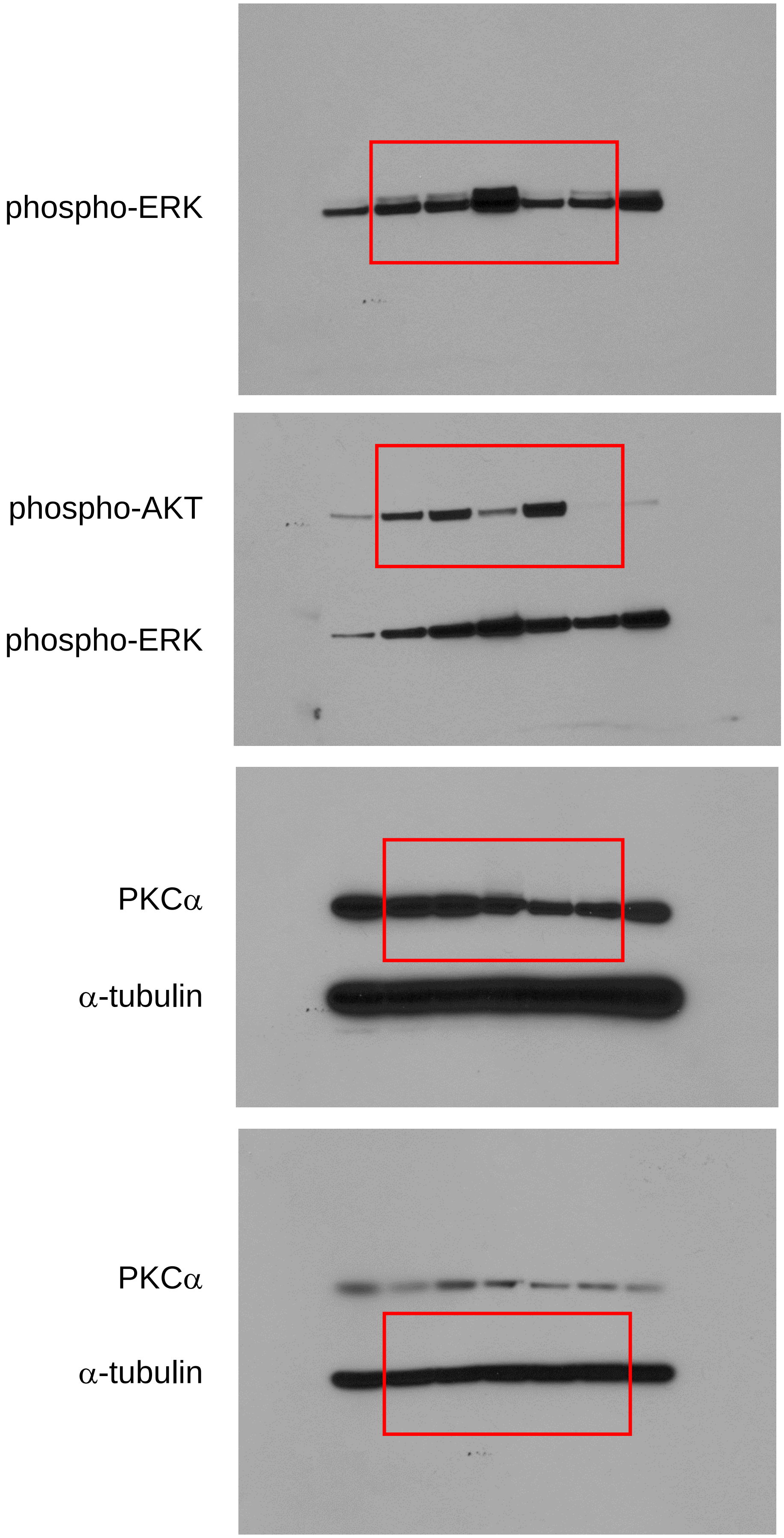


**Supplementary Figure 10.** Uncropped Western blots from Figure 2. The cropped region that is displayed in Figure 2b and 2c with sample labeling and molecular weight markers is highlighted in red for each blot.
