# Supplementary material for: A recurrent kinase domain mutation in PRKCA defines chordoid glioma of the third ventricle
Source: Nat Commun. 2018 Feb 23;9:810. doi: 10.1038/s41467-018-02826-8 (PMC5824822; doi:10.1038/s41467-018-02826-8)
Supplement: Supplementary file 3 — Description of Additional Supplementary Files [file 41467_2018_2826_MOESM3_ESM.pdf]

Description of Additional Supplementary Files

File Name: Supplementary Data 1
Description: Genes targeted for sequencing on the UCSF500 Cancer Panel in which no somatic nonsynonymous mutations, amplifications, deletions, or rearrangements were identified in the 13 chordoid gliomas. All coding exons were captured for sequencing from each of these 478 genes, with those highlighted genes also having select intronic or upstream regulatory regions that were captured for sequencing to enable detection of structural variants.

File Name: Supplementary Data 2
Description: Confirmed somatic nonsynonymous PRKCA mutations in the Catalogue of Somatic Mutations in Cancer (COSMIC) database, version 81 release.
